# Self-assembled silver nanoislands formed on glass surface via out-diffusion for multiple usages in SERS applications

**DOI:** 10.1186/1556-276X-7-676

**Published:** 2012-12-17

**Authors:** Valentina V Zhurikhina, Pavel N Brunkov, Vladimir G Melehin, Tommi Kaplas, Yuri Svirko, Victoria V Rutckaia, Andrey A Lipovskii

**Affiliations:** 1 St. Petersburg State Polytechnical University, Polytechnicheskaya 29, St. Petersburg, 195251, Russia; 2 Ioffe Physical Technical Institute of the Russian Academy of Sciences, Polytechnicheskaya 26, St. Petersburg, 194021, Russia; 3 University of Eastern Finland, Yliopistokatu 7, P. O. Box 111, Joensuu, 80101, Finland; 4 St. Petersburg Academic University - Nanotechnology Research and Education Centre of the Russian Academy of Sciences, Khlopina 8/3, St. Petersburg, 195220, Russia

**Keywords:** Silver island film, Reactive diffusion, Glass, SERS, 78.67.Sc, 81.16.Dn, 78.30.-j

## Abstract

We demonstrate that silver nanoisland film self-assembled on the surface of silver-containing glass in the course of thermal processing in hydrogen is capable to detect 10^−7^ M concentration of rhodamine 6G in water using surface enhanced Raman spectroscopy (SERS) technique. The film can be multiply restored on the same glass substrate via annealing of the glass in hydrogen. We showed that the film can be self-assembled after as much as ten circles of the substrate cleaning followed by annealing. The proposed technique of the silver nanoisland film formation enables multiple usage of the same glass substrate in SERS experiments.

## Background

Enormous sensitivity of the surface enhanced Raman spectroscopy (SERS) has attracted a lot of interest to the synthesis of metal island films (MIF) which are widely recognized as key elements of SERS-based sensors [1-5]. MIF are conventionally manufactured by depositing metals onto dielectric substrates using thermal and e-beam evaporation, sputtering, and chemical and plasma-chemical metal deposition [6]. The MIF performance in the SERS-based devices is mainly determined by the film morphology which can vary depending on the metal deposition technique as well as post-deposition processing.

Here we propose a new technique that enables MIF self-assembling on a dielectric substrate enriched with metal. The processing of such substrates in reducing hydrogen atmosphere results in the formation of the metal nanoislands on the substrate surface. This process can be described in terms of reactive diffusion of hydrogen, i.e., in terms of penetration of hydrogen into the substrate and its ionization via reducing metal ions to neutral atoms which are hardly soluble in glass and diffuse towards substrate surface. The accumulation of the metal atoms at the surface results in the formation of metal nuclei that grow and form metal nanoislands. We employed this technique to form silver island film on the surface of silver-containing phosphate glasses, and we characterized the morphology and optical properties of the MIF using atomic force microscopy, optical absorption, and Raman spectroscopy. In order to demonstrate the performance of the created MIF in SERS-based sensors, we showed that it is capable to detect 10^−7^ M concentration of rhodamine 6G (R6G) deposited on the glass surface. The developed technique does not require external metal source and enables multiple usages of the same glass substrate in SERS measurements.

## Methods

The phosphate glass with composition of 45P_2_O_5_-20Na_2_O-5K_2_O-10Ag_2_O-2Nb_2_O_5_-2B_2_O_3_-5ZnO-1ZrO_2_ and contains 10 mol% of Ag_2_O was synthesized in silica crucible at approximately 1,500°C. Island films were formed on the polished glass surface in the course of thermal processing in hydrogen atmosphere at 200°C. The hydrogen was produced by water electrolysis. The morphology of the manufactured MIF was characterized using Dimension 3100 (Veeco Instruments, Inc., Plainview, NY, USA) atomic force microscope (AFM) using probes with curvature radii below 10 nm.

In order to perform the SERS experiments, we deposited 10-μL drop of R6G aqueous solution on the substrate. After drying at room temperature, it formed a film spot with diameter of about 3 mm. The concentration of R6G in the solutions and surface concentration of the created R6G films are listed in Table 1. Note that the average surface concentration of R6G which is equal to 6 × 10^12^ cm^−2^ corresponds to 0.06 of the monolayer.

**Table 1:** Samples for SERS experiments

**Film name**	**R6G concentration in the solution (M)**	**Volume R6G concentration in the solution (molecules/cm**^**−3**^**)**	**Surface R6G concentration in the film (molecules/cm**^**−2**^**)**
R6G 10^−7^ M	10^−7^	6 × 10^13^	6 × 10^12^
R6G 10^−3^ M	10^−3^	6 × 10^17^	6 × 10^16^

Optical absorption spectra of the deposited R6G films were measured with UV–Vis Specord 50 spectrometer (Analytik Jena AG, Jena, Germany). Raman spectra at the excitation wavelength of 514.5 nm were obtained in backscattering geometry using Renishaw spectrometer (Renishaw PLC, Wotton-under-Edge, Gloucestershire, UK) with Raman microscope and charge coupled device detector. In the Raman measurements, the excitation power at the sample was equal to 700 μW, the diameter of the laser beam at the sample was approximately 10 μm, and the signal accumulation time was 10 s. Since the excitation wavelength coincides with the electron resonance transition in R6G molecule, hereafter, we will refer to this process as the surface enhanced resonant Raman spectroscopy (SERRS) [1] to emphasize the resonant enhancement of the measured SERS signal. The measurements were performed at room temperature 6 days after the films deposition.

## Results and discussion

### Island film formation and optical properties

**Figure 1:**
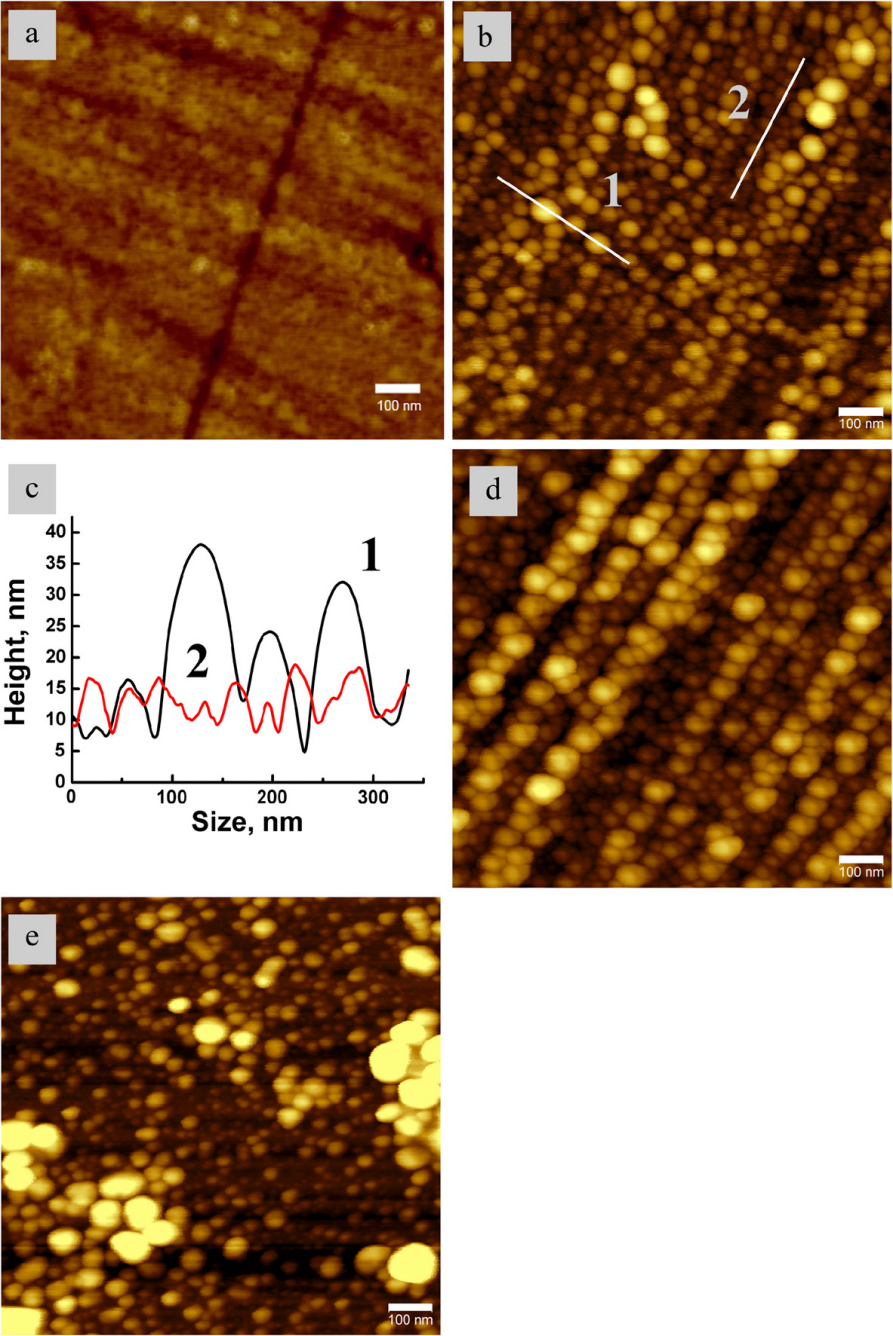
AFM images of the samples: (a) initial glass surface; (b) glass surface after the first anneal; (c) the profiles of bigger (1) and smaller (2) islands measured along lines 1 and 2 in image (b); the widths of maxima correspond to the particles size; (d) another region of the glass surface; (e) glass surface after 9 cycles of ‘removal-anneal’.

In the experiment, nanoisland films were formed on the glass surface after processing the sample in hydrogen. AFM images of the sample surface before and after 20-min thermal processing in hydrogen at 200°C are presented in Figure 1a,b. In Figure 1b, one can observe densely packed nanoparticles with diameters of about 60 nm on the glass surface. The shape of the nanoparticles is close to hemispherical or hemiellipsoidal, with longer axis perpendicular to the glass surface and wetting angle of about 90° (see Figure 1c).

Figure 1b,d shows that the centers of nanoparticles are aligned. Such an alignment is due to the microscratches created on the surface in the process of glass polishing. The surface defects associated with these microscratches become the nucleation sites giving rise to the long range order in the created island film [7]. It is worth noting that this phenomenon can be used to manufacture island films with prescribed geometry via formation of the required surface defects pattern on the glass surface.

The island film can be easily removed from the glass surface with e.g., water-wet cotton. It can be also transferred from the glass to a flexible substrate (e.g., scotch tape) that could be of interest for biomedical applications. After removal of the island film from the glass surface, e.g., by cleaning or transferring to a scotch tape), it can be easily restored by the thermal treatment of the sample in hydrogen atmosphere. Figure 1e shows the AFM image of the sample surface after 9 removal-anneal cycles. In this case we used mechanical cleaning of the glass surface with wet cotton.

**Figure 2:**
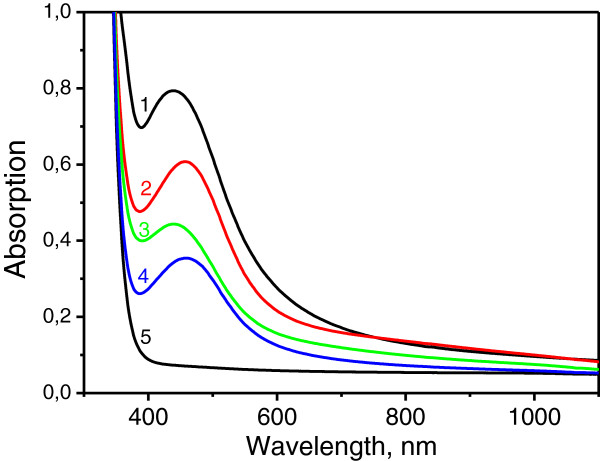
**Absorption spectra of the samples.** Curve 1, after the first anneal; curves 2 and 3, after 4 and 9 cycles of removal-anneal, respectively; curve 4, after 9 cycles of removal-anneal and final MIF removal; curve 5, after the first annealing and film removal.

Each annealing process at 200°C was 20 min long. Although particle size dispersion and packing density change after several annealing cycles (compare Figure 1d and e), our results demonstrate that the same silver-enriched glass substrate can be used in multiple SERS experiments. Figure 2 shows optical absorption spectra after several ‘anneal-removal’ cycles. One can readily observe from Figure 2 that the surface plasmon resonance (SPR) in silver nanoparticles [8] in the region of 450 nm dominates the spectra. The developed technique enables producing films of different morphology via varying both processing conditions (temperature and duration of the annealing) and number of removal-anneal cycles. Detailed studies of the dependence of the island film morphology on the processing mode will be presented in our forthcoming paper.

The process responsible for the formation of the MIF on the surface of metal-enriched glass is often referred to as the reactive diffusion. In this process, the diffusion of hydrogen ions into the glass is followed by the chemical reaction, in which silver ions bonded to non-bridging oxygen atoms are replaced by hydrogen [9]:

-O^−^-Ag^+^ + 1/2H_2_ → -O^−^-H^+^ + Ag^0^,

where O^−^ is non-bridging oxygen atom. This reaction results in changing of the glass composition in the subsurface region. The neutral silver atoms created in this reaction move towards the glass surface, nucleate, and finally form nanoislands of metal silver on the surface of the sample.

Optical absorption spectra of the silver nanoisland films formed using different number of removal-anneal cycles are presented in Figure 2. One can observe that the magnitude of SPR peak decreases with the number of annealing cycle and that the SPR disappears after cleaning of the glass surface. This experimental finding indicates that after the first thermal processing, silver nanoparticles are created on the surface of the substrate rather than in the bulk.

One can also observe from Figure 2 that the SPR ‘survives’ after 9 removal-anneal cycles (curve 3 in Figure 2). However, we found that after the final cleaning of this sample (curve 4 in Figure 2), the SPR peak remains in the absorption spectrum. This shows that the increasing reactive diffusion time results in the accumulation of silver nanoparticles not only on the glass surface, but also in the subsurface region of the phosphate glass. The modeling of the mechanism of the island film formation is presently in progress and it will be presented soon in our next paper.

It is necessary to note that in silicate glasses, the formation of nanoparticles on the surface was also both predicted [9] and experimentally observed [10]. However, in silicate glasses, the silver-sodium ion exchange and annealing in hydrogen result in silver nanoparticles growth predominantly in the subsurface layer [8]. In contrary, our results show that in phosphate glass, the main sink for neutral silver atoms is the glass surface. Thus, the formation of nanoisland silver films in the course of reactive diffusion is more efficient in phosphate rather than in silicate glass. Such a difference may originate from a higher solubility of neutral silver in phosphate matrixes in comparison with silicate ones.

### SERS studies

**Figure 3:**
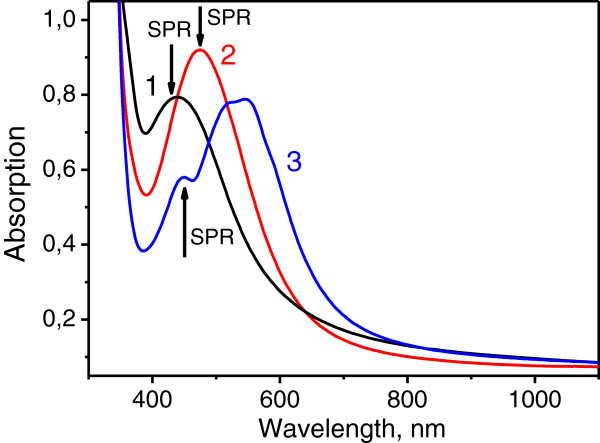
**Absorption spectra of the samples: curve 1, after the first anneal; curve 2, after the first anneal and formation of R6G 10**^**−7**^**M film; curve 3, after the first anneal and formation of R6G 10**^**−3**^**M film. The positions of SPR are marked with arrows.**

Figure 3 shows absorption spectra of the silver nanoisland films before (curve 1) and after the R6G film deposition (curves 2 and 3).

One can see from Figure 3 that at 10^−7^ M concentration of R6G, the R6G absorption resonance is not observable, while the SPR peak is red-shifted (compare curves 1 and 2). At 10^−3^ M R6G concentration, the R6G absorption resonance dominates the spectrum in 500 to 600 nm range, while the SPR peak remains at its initial position (compare curves 1 and 3). The reverse shift of the SPR may be due to the change in the R6G film composition and hence, the position of the electron resonance when the R6G concentration increases. Specifically, the detailed investigation of the silver MIF absorption spectra for different concentrations of deposited R6G performed by Zhao et al. [11] has shown that the thickness of the deposited R6G layer and its composition (fraction of monomers, dimers, and more complex aggregates) influences the shift, width, and amplitude of the SPR peak.

**Figure 4:**
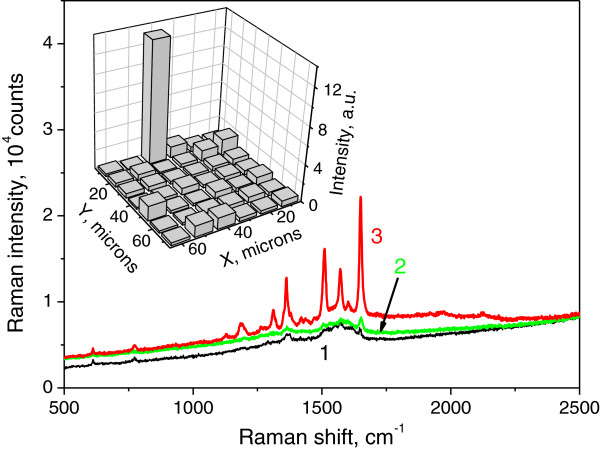
**SERRS spectra (R6G 10**^**−7**^**M film) measured at different positions in mapped area.** These positions correspond to 1,651 cm^−1^ line intensity below the average value (curve 1), above the average value (curve 2), and to ‘anomalous spot’ (curve 3). Inset: the SERRS signal mapping (at line 1,651 cm^−1^) for R6G 10^−7^ M film. Scan area, 60 × 70 μm^2^; step, 10 μm; vertical axis, intensity of the 1,651 cm^−1^ line in counts.

To study the performance of the manufactured MIF in SERRS, we performed Raman mapping of the R6G 10^−7^ M film with spatial resolution of 10 μm. Figure 4 shows the intensity distribution of the 1,651-cm^−1^ Raman line of R6G (with background subtracted) over 60 × 70 μm^2^ sample region. One can observe that the intensity of the SERRS signal is nearly the same over the whole scanned area except in one point, in which the intensity exceeds 15 times the average value. The regions where signal essentially exceeds the average one are called ‘hot spots’ [1]. The higher signal from the vicinity of the hot spot is due to the stronger enhancement of the local electromagnetic field by closely placed metal nanoparticles in comparison with an isolated metal nanoparticle. However, we have no evidence to suggest that the recorded signal is due to the hot spots because of the large area of the probing beam (10 μm) that should provide the signal about the same magnitude over the entire area of our scan [12]. It is more likely that the anomalously high signal is due to a surface defect where the R6G coating is thicker than the average. In Figure 4, we present SERRS spectra of the 10^−7^ M film measured in the regions of the scanned area shown in the inset. Curves 1, 2, and 3 represent Raman spectra measured in areas where 1,651-cm^−1^ line intensity was below, above, and anomalously high in comparison with the average value, respectively. One can see here that even without anomalous spot enhancement (curves 1 and 2), the signal/noise ratio in the SERRS spectra is high enough to detect 10^−7^ M R6G concentration.

## Conclusions

Hydrogen/metal ions reactive diffusion in phosphate glasses followed by transport of neutral metal to the glass surface enables formation of nanoisland silver films with the average island size of tens of nanometers. The dependence of the MIF morphology on the defects distribution on the glass surface allows one to create spatially structured island film of prescribed geometry. The developed technique can be employed to manufacture the films based on copper and other noble metals, and we believe that it will also find applications in SERS-based sensors in medicine and biology because it permits multiple usage of the same substrate in SERS measurements. We demonstrated in particular that MIF manufactured by the silver out-diffusion enables registering of 6 × 10^12^ cm^−2^ surface concentration of R6G molecules (0.06 of the monolayer) in the SERRS experiment.

## Abbreviations

AFM: Atomic force microscope; MIF: Metal island films; R6G: Rhodamine 6G; SERRS: Surface enhanced resonant Raman spectroscopy; SERS: Surface enhanced Raman spectroscopy; SPR: Surface plasmon resonance.

## Competing interests

The authors declare that they have no competing interests.

## Authors’ contributions

VZh searched the regimes of samples processing. PB carried out AFM measurements. VM performed SERS studies including mapping. TK dealt with the modification of measurements technique for SERS mapping and with optical absorption spectra measurements. YuS analyzed the optical absorption and SERS. VR prepared the samples from ion exchange until their annealing in hydrogen. AL supervised the whole work starting from sample preparation to analysis of data and edited the manuscript. All authors read and approved the final manuscript.
